# Needle guide in shoulder arthroscopy – a technique

**DOI:** 10.1186/1758-2555-1-7

**Published:** 2009-04-24

**Authors:** Sujith Sidharthan, Aloysius N Mbako, James R Wootton

**Affiliations:** 1 Department of Trauma & Orthopaedics, Maelor Hospital, Croesnewydd Road, Wrexham, Clwyd, LL13 7TD, UK

## Abstract

Distension of joint with fluid is often used to facilitate insertion of arthroscope. This may prove difficult at times in the shoulder joint, as unlike the knee, it is deeply situated, making extravasation of fluid outside the capsule, a common occurrence. This is especially true in very tight joints and is often a problem for beginners. We describe here a very effective and simple technique where a needle is used to distend the shoulder before the insertion of the arthroscope.

## Introduction

Unlike the knee joint, the shoulder is deeply situated with prominent muscular enclosure. Distension of the joint with saline is often used as a means to facilitate insertion of arthroscope. However, this may prove difficult at times especially in tight joints and for beginners. Inadvertent extravasation of saline into the soft tissues around the capsule may often occur as a result. Subsequent introduction of scope is difficult as the landmarks are obscured. A very effective and reliable technique to distend the shoulder joint is described here that can be used as a guide in the accurate placement of arthroscope in the joint.

## Technique

**Figure 1:**
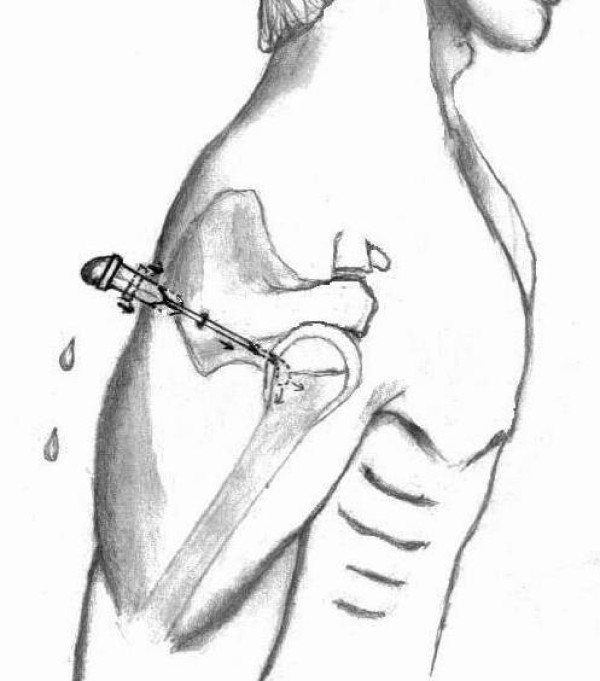
**Needle with the convex fluid film at its butt being advanced into the shoulder joint**. As the needle tip enters the shoulder joint, the fluid is sucked in.

Patient is positioned in either the lateral decubitus or the beach chair positions. Sufficient traction is employed to distract the joint. The traditional posterior portal [1,2] as popularised by Andrews is used. The entry point is placed above the midline of the glenoid approximately 1 cm medial and 1 to 2 cm inferior to the posterolateral corner of the acromion. This is the so-called 'soft spot' over the posterior aspect of the deltoid, approximating the interval between the infraspinatus and teres minor muscles. Note that the scapula is oriented 30 deg anterior to the coronal plane, which is the proper direction of entry. A 21 G hypodermic needle is inserted into the 'soft spot' posteriorly by aiming just lateral to or at the coracoid process, which is usually well palpated anteriorly. Needle is filled with fluid leaving a convex fluid film at the butt. The needle is slowly advanced in the direction of the shoulder joint (Figure 1). As soon as the needle enters the joint, the fluid in the needle is sucked in with a hissing ('hush') sound. Consequently, the convexity of fluid film at the butt of the needle is lost. This verifies the correct placement of the needle in the joint. Sometimes an audible 'hush' may not be present and the loss of convexity of fluid film with inward movement of the fluid may be the only sign evident. If the convexity of the fluid film is lost to reasons other than hitting the target, it may be recreated by refilling the butt and same technique repeated. Once confident of the placement, inject 5–10 cc of normal saline to distend the joint, and then disengage the syringe and look for free back flow of fluid from the needle to confirm placement in the joint. About 20 cc of fluid can be injected to fully distend the joint through the needle. The needle is then removed with a mental note of the direction of the needle. Skin incision is made at the position of the needle and the blunt trocar inserted in the direction of the previous needle. The right placement of the scope can be ascertained with a free back flow of the previously injected fluid in the joint through the scope.

## Discussion

The intra-articular pressure inside the shoulder is slightly negative [3]. It produces the suction effect that is encountered as soon as the needle enters the joint. This is further accentuated by the external traction employed at the joint. The technique provides a reliable means of locating the position of the joint even before actually distending the joint with the fluid. It nearly eliminates the possibility of distorting the landmarks with inadvertent injection of fluids in the soft tissues around the shoulder. The accurate position can be further verified by the free back flow of the fluid. The needle also provides a reliable means to fine-tune the direction of the portal entry. The subsequent placement of the arthroscope is made easier by the distended joint capsule. Once the joint is distended with fluid it widens the joint space and creates a cystic expansion of the redundant capsule. This provides a larger target with a firmer stretched capsule to aim at. That is why subsequent placement of the arthroscope is easier and more forgiving with the direction aimed.

## Conclusion

We believe the technique that has been described provides a reliable and accurate means to locate the shoulder joint and distend the capsule with fluid. Subsequent introduction of the arthroscope is made a lot easier. It is therefore a useful aid for beginners and in very tight joints.

## Competing interests

The authors declare that they have no competing interests.

## Authors' contributions

SS helped with the background research, picturised the procedure and wrote the paper with the assistance of the other authors. ANM helped assisted with preparation of the manuscript. JRW is the senior author, operated on all patients using the technique supervised and advised on the preparation of the manuscript.
